# Analysis of Heavy Metal Accumulation in *Ulva rigida* and its Effects on Seed Germination of *Pennisetum glaucum*

**DOI:** 10.1002/open.202400386

**Published:** 2025-01-28

**Authors:** Karungan Selvaraj Vijai Selvaraj, Rajiv Periakaruppan, Vanathi Palanimuthu, Valentin Romanovski, Ayyarappan Bharathi, Manu Mohan

**Affiliations:** ^1^ Vegetable Research Station Tamil Nadu Agricultural University, Palur 607102 Cuddalore India; ^2^ Department of Biotechnology PSG College of Arts & Science Coimbatore 641014, Tamil Nadu India; ^3^ Department of Biotechnology Sri Ramakrishna College of Arts & Science Coimbatore 641006, Tamil Nadu India; ^4^ Department of Materials Science and Engineering University of Virginia 22903 Charlottesville USA; ^5^ Department of Genetics and Plant Breeding Agricultural College and Research Institute Vazhavachanur 606 743, Tamil Nadu India; ^6^ Department of Chemical Oceanography Cochin University of Science and Technology Kochi, Kerala India

**Keywords:** *Ulva rigida*, Heavy metals, *Pennisetum glaucum*, Seed germination, Biofertilizer

## Abstract

The alga contains salt and heavy metals that are accumulated in algae poses a significant challenge to the safe use of algae in soil fertilization and other applications. This study examines the relevance of algal biomass as an environmentally friendly fertilizer, thereby contributing to sustainable coastal management practices. In this study, the hot and cold extraction method were done to obtain the *Ulva rigida* extract. Heavy metals such as vanadium, chromium, manganese, iron, nickel, cobalt, copper, zinc and cadmium etc. were analyzed using ICP‐MS. Heavy metal analysis showed that the major metals such as manganese, iron, vanadium and zinc in *Ulva rigida* extract. The algae extract was used in different concentration (20, 40, 60 and 80 μL) to analyze the seed germination study in *Pennisetum glaucum* and it was found that theseed germination were 100 % at 5^th^ day after sowing and the root and shoot length increased with increasing concentration of *Ulva rigida* extract and at 80 μL the shoot length of *Pennisetum glaucum* were decreased. The aqueous extracts of *Ulva rigida* are eco‐friendly, safe method for recycling the algal biomass as a novel biofertilizer.

## Graphical abstract

This study demonstrates the investigation of ***Ulva rigida*** seaweed extract as an eco‐friendly biostimulant and biofertilizer, combined with an analysis of heavy metal accumulation and its effect on ***Pennisetum glaucum*** (pearl millet) seed germination

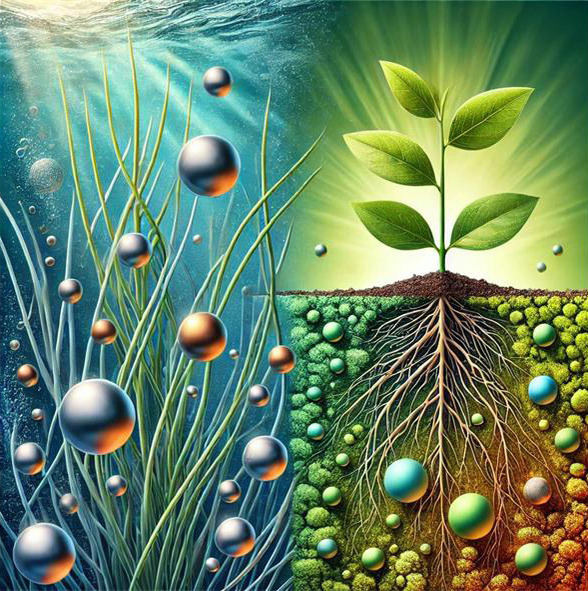

## 1 Introduction

A conventional agricultural practice such as the use of chemical fertilizers increases the availability of minerals and nutrients to plants and also increases the growth and development of plants. The utilization of huge quantities of chemical fertilizers causes negative effects including leaching, volatilization, decrease in soil microbiota, and decreased fixation of nutrients in soil and plants due to the accumulation of chemical fertilizers.^[1]^ Modern agriculture is looking for a new biotechnological approach to improve the nutrients to plants without affecting the growth, development, and yield of plants.^[2]^ Currently, the usage of organic food is increasing, and because of the demand for producing organic food, biostimulant processes are developing all around the world.^[3]^ It is a process of increasing the nutrients to plants and microorganisms and also adopting the plants to grow in stress‐tolerant conditions (Du Jardin, 2015).

Food and Agricultural Organization (FAO) 60 % reported that increase in food demand by 2050. Nowadays, seaweeds are, predominantly used in agriculture and horticulture research.^[4]^ Seaweed extract is one of the important biostimulants utilized for promoting the growth and uptake of nutrients by plants. Marine algae play an important role in inducing the growth and nutrient uptake of the plant due to the presence of potential bioactive compounds.^[5]^ Seaweeds contain numerous bioactive substances that can increase human health. Several algae have been authorized for human consumption because of the presence of more nutrients.^[6]^ The use of seaweed extract as a biostimulant is an eco‐friendly natural resource for improving seed germination, plant growth development, and nutrient uptake efficiency without any negative effects on plant growth and yield.[^5^, ^7^] The organic fertilizer derived from seaweed extract is non‐toxic, easily degradable in soil, no toxic intermediate compound will be produced, and tolerant to biotic and abiotic stress.[^8^, ^9^]

Seaweed extracts are produced by different extraction procedures for biofertilizer and biostimulant production from marine algae for agricultural practices. Most of the time, extracts are prepared by using water, alkalis, and acids, or by physical disruption of seaweed cells into fine particles.^[10]^ The most predominant method used for the production of seaweed extract is water extract.^[11]^ Thus, several researchers reported that the utilization of water extract from seaweed is predominantly used as a plant biostimulant to increase the growth and development of various plants such as wheat, tomato, spinach, Vigna sinensis, cereals, pulses, etc.^[12]^

The majority of green macroalgae consumed worldwide are *Ulva species*, commonly referred to as sea lettuce in the food industry. It belongs to *Ulvaceae* family. Ulva species are distributed widely based on climate, ecological, and opportunistic growth conditions that make them suitable and cost‐manageable for cultivation purposes. It is rich in polysaccharides, vitamins, minerals, protein content, and growth hormones such as IAA, cytokinin, and amino acids.[^13^, ^14^] Polysaccharides are a significant component of all seaweeds that constitute about 30–40 % of the extract. Algal polysaccharides used as a stimulant increase the growth of the plant.^[15]^ Several products were developed from the seaweeds, including sodium alginate from brown seaweeds, *Carrageenan* from red seaweed, and oligo‐carrageenans, all of which increased and stimulated the seed germination, growth, development, leaf biomass, chlorophyll content, and also enhanced the photosynthesis of plants such as *Verticillium wilt*, *Papaver somniferum L*, chickpea plants, maize, and *Pinus radiata*.[^16^, ^17^, ^18^, ^19^]

*Ulva rigida* is green algae and belongs to the *Ulvaceae* family. It is growing near the submerged marine rocks in various seas and oceans around the globe. The most important constituents of *Ulva rigida* are polysaccharides, proteins, ashes, and liquids. Starch and cellulose are the most important polysaccharides present in *Ulva rigida*. Apart from that, this alga also contains polysaccharides such as ulvan, which contains xylose, glucuronic acid, sulfate esters, and rhamnose.^[20]^

Pearl millet (*Pennisetum glaucum*) is the sixth most important cereal globally and the fourth most important tropical cereal. It is grown in Asia (11 million hectares annually), Africa, and Brazil (2 million ha annually). It is cultivated in low fertility, light textured soil with receiving less than 500–600 mm rainfall areas. It is an annual grass, erect, and reaches up to 2 m tall.[^21^, ^22^]

This current research work is focused on investigating the effects of *Ulva rigida* extract as a biostimulant for seed germination, shoot, and root formation of *Pennisetum glaucum*.

## Materials and Methods

### Collection of Algae and Chemicals

*Ulva rigida* was collected from Mandapam, Ramanathapuram district,Tamil Nadu, India. Chemicals and reagents with 99.5 % purity and glassware were obtained from Sigma‐ Aldrich, Mumbai.

### Preparation of Aqueous Extract

*U. rigida* was carefully taken and washed under running tap water to remove the unwanted sand particles in the sample. The excess sample was washed thoroughly and sliced into small pieces. The sliced pieces of the sample were stored in a polythene bag at −20 °C for further use.

### Preparation of Hot Extract

5 g of *U. rigida* was taken and ground using a mortar and pestle using distilled water. Then the extract was filtered through Whatman No. 1 filtered paper. The filtered sample was made up of 100 mL of distilled water and boiled in a water bath for 30 mins at 100 °C.

### Preparation of Cold Extract

5 g of *U. rigida* was taken and ground using a mortar and pestle using 100 mL aqueous extract. It was centrifuged at 5000 rpm for 5 min. The resultant supernatant was collected and filtered through the Whatman No. 1 filter paper. Later, the extract was stored at 4 °C until further use.

### Analysis of Heavy Metals

The prepared hot and cold aqueous extract of *U. rigida* was used to determine the presence of the following minerals: vanadium, chromium, manganese, iron, nickel, cobalt, copper, zinc, cadmium, arsenic, selenium, and lead using inductively coupled plasma mass spectrometry (ICP‐MS).

### Seed Germination Assay

The seeds of Pennisetum glaucum were collected from Tamil Nadu Agricultural University located in Coimbatore; Tamil Nadu. The seeds of the same size were surface‐sterilized in a sodium hypochlorite solution (10 %) for five minutes. Following this, seeds were washed three to five times with distilled water. Ten seeds were sterilized and soaked in U. rigida extract (hot extract), and then placed on filter paper in 9 cm sterile Petri dishes. Different concentrations (20, 40, 60, and 80 μL) of *U. rigida* extract were poured into the seeds. The positive control was distilled water. Each plate was covered with parafilm to stop infection and solution evaporation. Laminar flow was used for all processes. The Petri dishes were given labels and kept in a germinator with a temperature of 25 °C and 18/6 hours of day and night light. Up until the end of the 5th, the calculation of germinated seeds was noted. The germination percentage and shoot and root length were calculated.

Germinationpercentage(%)=100·n/N.

where n is a number of germinated seeds on the 5th day and N is a number of seeds.

### Statistical Analysis

All the analysis was carried out with three replications. The obtained results and data were analyzed using the SPSS software and results were expressed as Mean±SD.

## 2 Result and Discussion

### 2.1 Analysis of Minerals and Heavy Metals in Hot and Cold Extraction of *U. rigida* by ICP‐MS

According to the ICP‐MS analysis, elements such as vanadium, chromium, manganese, iron, nickel, cobalt, copper, zinc, cadmium, arsenic, selenium, and lead were detected in *U. rigida*‐hot and cold extract. Results of the concentration of the minerals in *U. rigida*‐hot extract and cold extract are given in Table 1. Generally, a significant difference was noticed between both the extracts of seaweeds and was found that the concentration of heavy metals and minerals in the hot extract of *U. rigida* was considerably higher than compared to the cold extract process of *U. rigida*. The concentration of vanadium was higher in hot extract of *U. rigida* (65.87 mg/kg) than compared to the *U. rigida*‐cold extract (68.55 mg/kg). The hot extract of *U. rigida* has an increased concentration of chromium (173.19 mg/kg), manganese (108.16 mg/kg), Iron (164.87 mg/kg), nickel (4.20 mg/kg) than compared to *U. rigida*‐cold extract. The trace amounts of cobalt, selenium, and cadmium were present in both hot and cold extracts of *U. rigida* (Table 1). The content of zinc (71.31 mg/kg) was high in the hot extraction process, and in the cold extraction process, the content of zinc (11.68 mg/kg) was reduced. Moreover, the mineral and heavy metal content of seaweed varied depending on the monsoon and season. Their ability to absorb and accumulate mineral content was 100 times higher than the of terrestrial plants.^[23]^ Čmiková et.al.,^[24]^ reported that the content of elements including copper, cobalt, chromium, iron, manganese, selenium, and zinc was analyzed using ICP OES 720 and was found to be higher in seaweed extracts than compared to land plants, and they have high antioxidant, antimicrobial properties. Truus et al.^[25]^ analyzed the elemental composition such as Pb, Cd, Zn, Cu, and Cr from the brown algae *Fucus vesiculosus*. Queiróset al.^[26]^ determined that trace elements such as iron (35.7–170 mg/100 g), zinc (0.8–1.9 mg/100 g), manganese (2.6–11.5 mg/100 g) concentration are higher in *U. rigida*. The difference in the accumulation of these elements between the two extraction processes is due to the conditions such as surrounding water, light, temperature, and uptake capacity of seaweed.^[27]^

**Table 1:** Elemental analysis in *U. Rigida* hot and cold extraction.

Heavy metals	*U. rigida ‐* hot extract (μg/g)	*U. rigida ‐* cold extract (μg/g)
Vanadium	65.87±0.02	68.55±0.05
Chromium	173.19±1.12	8.55±0.02
Manganese	108.16±2.42	104.03±2.25
Iron	164.87±2.58	80.22±2.36
Nickel	4.20±0.25	0.00
Cobalt	0.83±0.01	0.51±0.01
Copper	11.49±0.12	24.35±0.16
Zinc	71.31±1.23	11.68±0.25
Cadmium	0.88±0.10	0.54±0.12
Arsenic	9.17±0.01	15.88±0.02
Selenium	4.43±0.012	0.0
Lead	0.0	0.0

### 2.2 Seed Germination Assay

The result presented in Table 2 indicates the application of *U. rigida* extract on the seed (*Pennisetum glaucum*) which shows the positive effect by stimulating the germination of the seed (*Pennisetum glaucum*). It revealed a significant difference between the experimental treatments (20, 40, 60, and 80 μL) with seaweed extracts. 100 % of the seeds were germinated at different concentrations of *U. rigida* extract and triplicates were maintained. The germination percentage, root length, and shoot length were found to be higher in all the treatments than compared to the control. The root and shoot length on the 5^th^ day in control was found to be 3±0.2 cm and 8±1.2 cm. The root and shoot length were increased when the concentration of *U. rigida* extract was increased. The root length was the same in all the treatments, but the shoot length of *Pennisetum glaucum* was high at 60 μL of *U. rigida* extract (11±2.5 cm) and it decreased when the concentration of *U. rigida* extract (80 μL) increased (10±1.8 cm). Mamede et al.^[28]^ reported that the seaweed polysaccharides extracted from *Saccorhizapolyschides*, *Gracilariagracilis* and *Chondruscrispus* increased the seed germination (98.67 %) of *Brassica napus* L. Thriunavukkarasu et al.^[29]^ stated that the *Padina gymnospora*, *Gracilaria edulis* and *Ulvafasciata* seaweed aqueous extracts were used as seaweed biofertilizer for the germination of *Capsicum annuum*. The seeds were soaked in different concentrations of seaweed extract, and the seed germination percentage of *Capsicum annum* was found to be 75 %. Sasikala et al.^[30]^ investigated that the soil fertility, seed germination, growth, and yield of *Solanum lycopersicum* were increased when *Sargassum tenerrimum* seaweed extract was used as a biofertilizer.

**Table 2:** Impact of *U. rigida* extract on the seed germination of *Pennisetum glaucum*.

Treatments	5^th^ day		
	Seed Germination (%)	Root length (cm)	Shoot length (cm)
Control	100	3±0.2	8±1.2
20 μL of *U. rigida* extract	100	4±0.7	9±1.0
40 μL of *U. rigida* extract	100	4±1.3	10±2.0
60 μL of *U. rigida* extract	100	4±1.5	11±2.5
80 μL of *U. rigida* extract	100	4±1.2	10±1.8

Heavy metals can have a dual effect on plant growth, acting as both biocatalysts and toxicants, depending on their concentration.[^31^, ^32^, ^33^] In low doses, elements such as zinc, copper, iron, manganese and cobalt play a key role in the biochemical processes of plants. They participate in metabolic pathways such as photosynthesis, chlorophyll synthesis, and enzymatic reactions.^[34]^ For example, iron is necessary for protein synthesis and the functioning of the electron transport chain in photosynthetic reactions, and zinc is actively involved in protein synthesis and the regulation of enzymatic activity. A number of researches proved this.[^35^, ^36^, ^37^, ^38^, ^39^, ^40^] Thus, the presence of these metals in algal extracts such as *Ulva rigida* can stimulate plant growth by increasing nutrient uptake and root development. However, with increasing dosage of metals, as was noted in the concentration of 80 μL extract, their effects can become toxic to plants. Exceeding threshold levels of heavy metals such as zinc, copper or iron can lead to inhibition of shoot growth, as was observed in experiments with *Pennisetum glaucum*. High concentrations of these metals cause oxidative stress, damage cell membranes and disrupt normal metabolic processes in plants. For example, excess iron can lead to the formation of reactive oxygen species, causing damage to cellular structures. Also, excessive accumulation of metals can disrupt the balance of macro‐ and microelements in plant tissues, which leads to weakened growth and reduced productivity. Thus, although metals can act as biocatalysts that promote plant growth, their concentration must be strictly controlled. Exceeding the optimal dosage, as demonstrated with 80 μL of *Ulva rigida* extract, leads to a decrease in the effectiveness of growth stimulation and deterioration of the plant.

While Ulva rigida extracts show promise as biofertilizers due to their ability to enhance seed germination and plant growth, potential challenges and limitations should also be considered. One significant concern is the accumulation of heavy metals such as cadmium (Cd) and arsenic (As), which are toxic to both plants and soil ecosystems when present above permissible levels. Based on the study, the concentrations of Cd and As in the U. rigida extracts were measured as 0.88 μg/g (hot extract) and 9.17 μg/g (cold extract) for Cd, and 15.88 μg/g (cold extract) for As. These levels highlight the importance of monitoring and controlling their concentrations in agricultural applications. According to environmental guidelines, the maximum permissible concentrations (MPCs) for Cd and As in soil are typically 3 mg/kg and 20 mg/kg, respectively, depending on regional regulations. In plants, the MPCs for Cd and As are usually 0.1 mg/kg and 1.0 mg/kg (dry weight). Exceeding these thresholds can lead to bioaccumulation in crops, posing risks to human and animal health through the food chain. Furthermore, excessive heavy metals can negatively impact soil microbial activity, reducing soil fertility and nutrient cycling. To mitigate these risks, the following measures are recommended: i) conduct routine assessments of Cd and As levels in both U. rigida biomass and treated soils; ii) optimize extract concentrations to ensure heavy metal levels remain within safe limits for agricultural use; iii) harvest U. rigida from uncontaminated waters to minimize the initial heavy metal content. Despite these challenges, careful application and adherence to safety guidelines can allow the benefits of U. rigida extracts to be harnessed sustainably while mitigating potential drawbacks. Further studies on extraction methods and long‐term impacts on soil and plant systems are also warranted.

### 2.3 Recommendations for further Research

Future studies in this area should prioritize addressing the presence of cadmium and arsenic in the extracts to ensure food safety and compliance with regulatory standards. Detailed toxicological assessments and bioaccumulation studies are necessary to confirm that the use of these extracts does not result in harmful accumulation of these elements in edible plant parts. Researchers should explore selective elimination techniques, such as employing microbial biomass, including bacteria like Bacillus subtilis or fungi like *Aspergillus niger*, as biosorbents to remove cadmium and arsenic. Chelating resins with specific functional groups, such as iminodiacetic acid, could also be investigated for their high selectivity in adsorbing these metals. Cultivating hyperaccumulator plants, such as *Pteris vittata* for arsenic and *Thlaspi caerulescens* for cadmium, before planting could help reduce soil contamination. Optimizing pH‐controlled extraction processes should be considered, as the solubility of cadmium and arsenic varies with pH, potentially minimizing their extraction while preserving beneficial components. Additionally, the use of nanomaterials, such as iron oxide nanoparticles with high adsorption capacity for these elements, warrants exploration. Blending strategies could be employed to dilute the concentration of cadmium and arsenic by combining *Ulva rigida* extract with other seaweed extracts containing lower levels of these elements. Throughout this process, rigorous testing and toxicological evaluations at each stage are essential to ensure that the final biofertilizer product is both safe and effective, with cadmium and arsenic levels in target crop plants remaining below regulatory limits. By adopting these approaches, researchers can contribute to the development of sustainable and safe biofertilizer products that align with food safety standards. Future work could also explore the application of machine learning techniques to analyze the large dataset generated from these experiments, allowing for predictive modeling of material performance under various conditions.[^41^, ^42^, ^43^] In increasingly complex agro‐climatic conditions[^44^, ^45^, ^46^, ^47^, ^48^, ^49^] for growing agricultural crops, the use of elements of the circular economy is especially relevant.[^50^, ^51^]

## 3 Conclusions

It was found that *Ulva rigida* extracts showed 100 % germination of *Pennisetum glaucum* seeds and increased the length of roots and shoots at different concentrations. However, at higher concentrations, such as 80 μL, a decrease in shoot length was observed. During the analysis of heavy metals by ICP‐MS, elements such as vanadium, chromium, manganese, iron and zinc were detected in the extracts, which confirms the possibility of using these extracts as biofertilizers. The data indicate the prospects for the use of *Ulva rigida* extracts in agriculture to increase the yield of various crops, as well as the utilization of algae as an environmentally friendly resource.

## Ethical Approval

Not applicable.

## Funding

Not applicable.

## Author Contributions

Karungan Selvaraj Vijai Selvaraj: investigation, formal analysis, validation, data curation, writing‐original draft; Rajiv Periakaruppan: conceptualization, supervision, investigation, formal analysis, validation, data curation, writing‐original draft, writing‐review and editing, project administration; Vanathi Palanimuthu: formal analysis, validation, data curation, investigation. Valentin Romanovski: formal analysis, validation, data curation, writing‐original draft, writing‐review and editing. Ayyarappan Bharathi: formal analysis, validation, data curation, investigation. Manu Mohan: formal analysis, validation, data curation, investigation.

## Conflict of Interests

The authors declare no competing interest with any previous work.

## Data Availability

All data, models, and code generated or used during the study appear in the submitted article.
